# Cutting Forces and Tool Wear Investigation during Turning of Sintered Nickel-Cobalt Alloy with CBN Tools

**DOI:** 10.3390/ma14071623

**Published:** 2021-03-26

**Authors:** Wojciech Zębala, Grzegorz Struzikiewicz, Ksenia Rumian

**Affiliations:** Chair of Production Engineering, Faculty of Mechanical Engineering, Cracow University of Technology, Al. Jana Pawła II 37, 31-864 Kraków, Poland; wojciech.zebala@pk.edu.pl (W.Z.); ksenia.rumian@pk.edu.pl (K.R.)

**Keywords:** machining, CBN, sintered nickel-cobalt alloy, specific cutting force

## Abstract

This article describes issues related to the machining of parts made of sintered nickel-cobalt alloy. Longitudinal turning with a CBN (cubic boron nitride) tool was analyzed. The results of experiments showed the influence of cutting parameters in the field of finishing machining on the values of cutting forces and specific cutting force, taking into account the wear of the cutting edge. Measurements and analysis of the topography and roughness parameters of the machined surface, as well as the cutting tool wear, were presented. The microscopic examination showed that the average grain size of the sintered nickel-cobalt alloy was 3.22 ± 0.1 (μm). The presence of the hardening state variability of the material during machining, as well as the value of the cutting force fluctuation as a function of the tool wear *VB,* were stated. The specific cutting force values increased to a small degree for the tool wear in the range of *VB* = 0–0.2 mm, and reached similar values in the range *k_c_* = 5500–7500 N/mm^2^. The specific cutting force values increased significantly for wear *VB* > 0.2 mm and were characterized by a large variability. The occurring phenomena were analyzed and several explanations were proposed. A recommendation was developed for the machining of parts made of sintered nickel-cobalt alloy. The Taguchi method was used in the experiment methodology.

## 1. Introduction

Nickel-cobalt based alloys are mainly used in the aviation industry for the production of engine parts [1,2]. These kind of materials are intended mainly for operation at high temperatures in parts such as turbine blades, engine housings, etc. [3,4]. The most important features of nickel-cobalt based alloys include: high hardness, tendency to react with the workpiece (formation of built-up edges on the cutting edge), high strength at high temperature, low thermal conductivity, and the ability to harden in the cutting zone, which makes these alloys tend to harden in the top layer [5,6]. Thellaputtaa et al. [1] and Deng et al. [2] stated that the characteristic properties of such alloys are a relatively high resistance to oxidation, corrosion resistance in an aggressive environment, resistance to thermal fatigue and creep, and good mechanical properties, both at high temperatures, as well as at cryogenic temperatures [7,8].

Currently, the main focus of research is on the surface quality and the integrity of the surface layer of elements made of the nickel-cobalt alloy. This is important for the durability, functionality, and fatigue limit of the processed elements [9,10]. Various criteria are considered during the optimization of machining of such materials, e.g., surface roughness, maximization of the tool life, and minimization of production costs [11]. Pawade et al. [12] proposed an approach to optimize high-speed turning with the use of TGRA (Taguchi gray relation analysis). Taking into account a significant number of limitations in the cutting process (e.g., related to a machine tool, a workpiece, and the required condition of the surface layer of the material), the problem of selecting optimal cutting parameters is very complex, as demonstrated by Pawade et al. [12]. An analysis of stresses and deformation during the cutting process was performed using the finite element method and verified experimentally by Peng et al. [13]. Struzikiewicz et al. [14] used the TGRA method to optimize the cutting process and to select the optimal parameters for turning sintered alloys made by a laser sintering method.

Nowadays, it is more and more popular to produce parts from metal powders. These materials have a different microstructure than that obtained by casting or forging [15]. The microstructure resulting from the production of an alloy from metal powders has been investigated by various authors. Krawczyk et al. [16] compared two materials: one prepared with casting and another with an additive SLM (Selective Laser Melting) method. They investigated how easily the materials could be machined and analyzed their structure. They demonstrated that during face turning for higher feed ranges, the quality of the machined surface was better for the cast material. Araujo et al. [17] found that with appropriate thermomechanical treatment it is possible to obtain optimal strength of cobalt-nickel alloy with a fine-grained structure while maintaining its properties. Nayan et al. [18] investigated the behavior of nickel-cobalt alloy during hot deformation in order to understand the evolution of the microstructure as a function of strain rate and temperature. This study revealed dynamic recrystallization, occurring at higher material processing temperatures. Based on the research conducted by Wang et al. [19] and DebRoy et al. [20], it can be concluded that the nickel-cobalt alloy obtained using this method has a fine-grained structure, and depending on the applied heat treatment, the material may have increased strength, while reducing plasticity, and it is possible to remove residual stresses and displacements occurring in material. The microstructure depends on the chemical composition and heat treatment. This nickel-cobalt alloy is a complex material intended for processing in AM (additive manufacturing) processes, because it contains a large number of alloy elements and is a precipitation hardened alloy [21,22].

Nickel-based superalloys are known as difficult to machine materials [23]. The authors described the current state of knowledge about the machining characteristics of nickel-based superalloys, explained the influence of different cutting parameters, the cooling method, and the type of cutting tool on the surface integrity features of the material after machining (surface roughness, defects such as surface defects, cracks, feed marks, grooves). Jawahir et al. [24] focused on presenting experimental and theoretical research on the integrity of surfaces in cutting processes, such as residual stress, hardness, and roughness. According to Chen et al. [25], changes in surface and subsurface zones demonstrated during the above-mentioned studies may significantly deteriorate the efficiency and shorten the fatigue limit of the processed parts. The formation of a white layer on the surface and in the machining zone, according to Ezugwu et al. [26] and Devillez et al. [27], is a typical structural change caused by machining in a nickel-cobalt alloy. During high-speed machining of a cobalt-nickel alloy [28], factors influencing the surface roughness are built-up edge, adhesion, and feed marks. Sadat et al. [29] showed the lesions resulting from machining with a tool made of tungsten carbide. These lesions included dents, grooves, cracks, and microcracks. In addition, Sharman et al [30], Zhou et al. [31] and Zhou et al. [32] showed that the built-up edge is the main cause of tool damage, the increase in surface roughness, and the greater the wear of the tool. The use of coolant causes less surface damage [30].

Gupta et al. [33] conducted research on nickel-cobalt alloy, while taking into account various cooling methods and observed that the MQL (minimum quantity lubrication) method is a better cooling technique compared to dry and flood cooling.

Many scientists have studied the residual stresses in machined surfaces and the effect of turning process parameters on the quality of the produced parts. Hua et al. [34] investigated how the direction and value of the main residual stress influences the fatigue limit of the turned nickel-cobalt alloy. They showed that the value of the main residual stress is much greater than the residual surface stress, and that it increases with increasing feed. They found that the main residual stresses can be the main indicator for assessing the effect of the residual stress on the fatigue properties of the turned alloy material. Pawade et al. [35] and Arrazola et al. [36] investigated the residual stresses along the tangential direction of the machined surface when turning nickel-cobalt alloy at high speeds. They showed that the tensile residual stresses increased with the increase of the cutting speed. With the increase in feed, the compressive residual stress changed to tensile stress. Hua et al. [37] investigated the effect of cutting speed, feed, and tool nose radius on the roughness, microhardness, and the degree of hardening of a nickel-cobalt alloy material surface during dry turning. They showed that the feed speed and the nose radius of the tool have the greatest impact on the roughness of the machined surface, while a higher cutting speed and feed increase the material hardening. They concluded that a larger nose radius significantly reduces the degree of material hardening.

D’Addona et al. [38] investigated the efficiency of the machining of a nickel-cobalt alloy at high cutting speeds. They used surface quality after machining and the wear of the cutting tool as the preferred parameters for the analysis of the cutting performance. They showed that with the increase of the cutting speed, the tool wear was faster.

Many authors have analyzed the influence of parameters such as feed, cutting speed of the tools on the microhardness, and roughness of the machined surface of the material in the turning process [39,40].

Unfortunately, despite many studies having been carried out by scientists, the problem of dedicated procedures for optimizing the machining of sintered materials has not been studied in detail, in particular for sintered nickel-cobalt alloy. The authors decided to fill this gap and made an attempt to establish a procedure for determining the optimal cutting parameters of sintered nickel-cobalt alloy, while minimizing the value of the cutting resistance, and taking into account the quality of the machined surface. The article presents the results of measurements of the cutting tool wear and surface roughness, as well as an analysis of topography and microscopic photos of the machined surface, cutting edge, and chip. The obtained results were verified by comparing the test results for the cast nickel-cobalt alloy with similar properties.

This article is organized as follows: The introduction is included in Section 1, Section 2 contains the description of the applied materials, research plan, and the test bench. This section also includes characteristics of the structure and composition of the material before turning. The case study is described in Section 3, where all research results for the applied material are discussed and presented. Conclusions are presented in Section 4.

## 2. Materials and Methods

In order to conduct the tests, material samples were prepared as a ring with an outer diameter *Dc* = 330 mm, manufactured with the alloy powder based on nickel and cobalt using a laser sintering method (AM250 Renishaw, Kingswood, New Mills, UK). A series of machining tests were conducted for longitudinal turning of the outer surface of the ring. As part of the research, wear measurements of the cutting edge were carried out with three machining speeds *v_c_* = 160, 180, and 200 m/min, and with constant values of feed and depth of cut. The state of the cutting edge was determined by measurement of the maximum value of the *VB* parameter (the flank wear of the cutting edge) on the flank surface. In addition, the values of the total cutting force components (cutting force *F_c_*, feed force *F_f_*, and thrust force *F_p_*) were obtained during cutting, and the *Ra* parameter value describing the roughness of the machined surface for each machining test was measured. Average values of 3–5 measurements were determined for each experiment. All turning runs were performed with a cubic boron nitride (CBN) cutting insert.

The following machining test conditions were adopted:

- cutting speed: *v_c_* = 160, 180, 200 m/min
- feed: *f* = 0.048 mm/rev
- cutting depth: *a_p_* = 0.5 mm
- dry machining

Machining tests were carried out on a test bench which included:

- Masterturn 400 lathe (KNUTH Werkzeugmaschinen GmbH, Wasbek, Germany),
- piezoelectric dynamometer type 9257B, with a Kistler 5070A amplifier (Kistler, Winterthur, Switzerland) and DynoWare software (Version 2825A, Kistler Group, Winterthur, Switzerland),
- test bench equipped with an Intra 50 profilometer by Taylor Hobson (Leicester, UK) for measuring surface roughness
- Jeol JSM 6460LV (JEOL Ltd., Tokyo, Japan) microscope for examining the microstructure by scanning electron microscopy.

**Figure 1:**
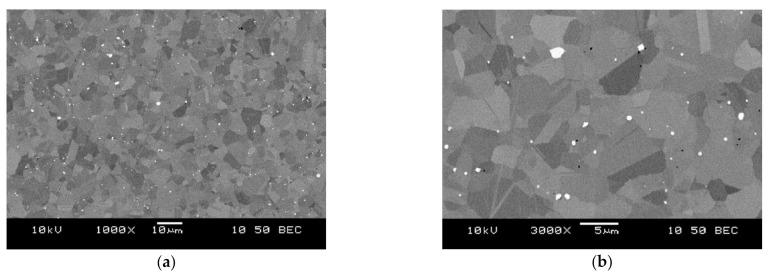
Microstructure of the workpiece material used in the current study before machining (sintered material) at magnification 1000× (**a**) and 3000× (**b**).

**Figure 2:**
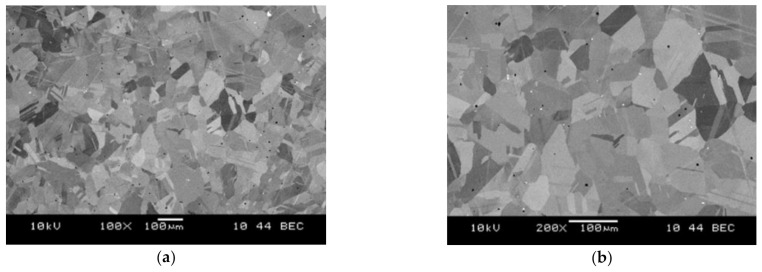
Microstructure of cast alloy at magnification 100× (**a**) and 200× (**b**).

Figure 1 shows the microstructure of the workpiece before the cutting process. The magnifications 1000×, 2000×, 3000×, and 5000× were used for analysis of the microstructure of the metallographic microsections which were prepared on the surface of the material to be machined. In addition, the images were recorded in the mode which presents the differences in the chemical composition: BEC (Battery Eliminator Circuit). The microstructure showed small, bright, round grains and larger ones with irregular shapes, with different shades of gray. The micro-area analyses’ aim was to determine the chemical composition of individual grains. These analyses mainly showed the presence of increased contents of molybdenum, chromium, and tungsten in round, bright particles. The analyses of the remaining grains, similar in shape, and with different shades of gray, did not show any significant differences in their chemical composition. The difference in shade was a result of a different crystallographic orientation, but the grains had the same phase, which was also visible in the surface distribution of the elements. In order to determine the differences in microstructure in different areas of the sample, measurements of the average grain size were made in the area at the edge of the sample and in the middle. Determination of the mean grain size was made stereologically, using the secant method. For the measurements, electron images of the microstructure were taken in the BEC backscattered electron mode at 1500× and 2000× magnification. The results of the mean grain size calculations are presented in Table 1. In order to compare the results, similar measurements were carried out for the material made by casting. Figure 2 shows the microstructure of the cast alloy.

The measurements showed that the tested material contained grains with an average size of 3.2–3.4 μm. The grain size in the examined areas of the sample did not differ significantly, and the material was homogeneous in terms of grain morphology. This was confirmed by the fact that the material was obtained by sintering from powders. The measurements carried out for the cast alloy showed that the tested material had an average grain size of around 35 μm. It should be noted that this value is 10 times greater than that of the sintered material.

Moreover, an analysis of the chemical composition was performed along a line leading from the edge of the sample to its center. Changes in the concentration of individual elements resulting from passing through grains of different chemical composition were registered. No significant changes were observed in the content of elements related to the line penetration into the material. In order to determine the chemical composition of individual grains, as well as to determine the average chemical composition of this material, analyses were performed in micro-areas using the EDS (Energy Dispersive X-ray Spectroscopy, JEOL Ltd., Tokyo, Japan) method. The analysis of the chemical composition of the entire area visible at 2000× magnification is presented in Table 2.

In the cutting tests, cutting edges with a corner radius *r_ε_* = 0.8 mm were made of CBN. The tool geometry is presented in Table 3.

**Figure 3:**
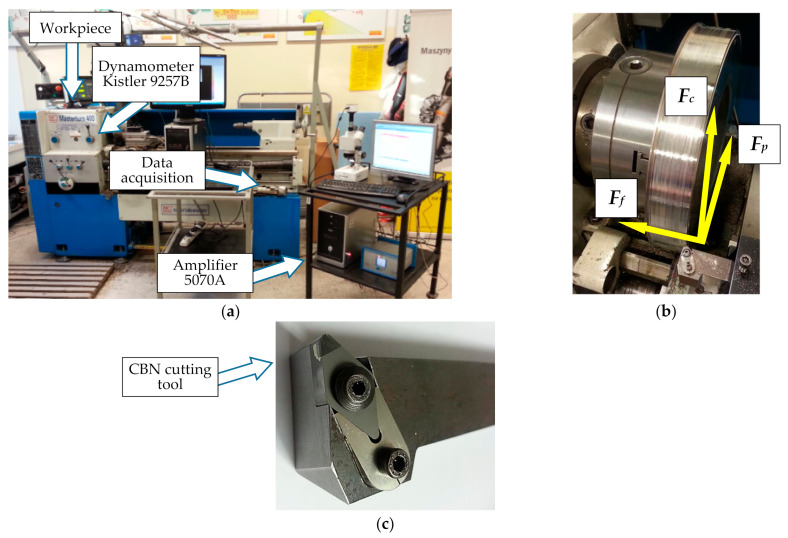
Test bench (**a**), fastening of the workpiece (**b**), and cutting tool with cubic boron nitride (CBN) insert (**c**).

The cutting parameters values were within the range of the finishing cutting parameters recommended by the tool manufacturer for nickel-based materials (S group). Figure 3 shows photographs of the test bench, cutting tool, and workpiece.

**Figure 4:**
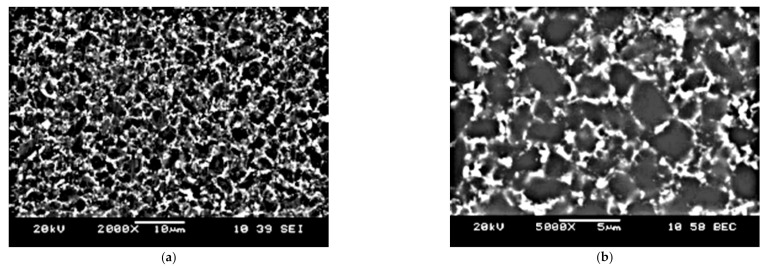
SEM micrograph of the CBN tool: (**a**) magnification 2000×, (**b**) magnification 5000×.

The next stage of the experiments was to determine the microstructure and chemical composition of the material used for the cutting edge. For this purpose, the lateral surface of the cutting insert was polished, on which the microstructure and chemical composition analysis was carried out. The tests showed that the cutting material was made of a bimodal sinter of cubic boron nitride with a cobalt binding phase (10% mass). EDS analysis also showed a low content of elements such as aluminum (1.5%), nickel (0.9%), and tungsten (2.8%, which most probably came from the process of homogenizing the mixture before sintering the cutting edge). Figure 4 shows the microstructure of the cutting edge surface.

The research plan was developed according to the Taguchi method [13]. Based on the analysis of the problem, the research plan was adopted in accordance with Table 4. The influence of two variables, cutting length (*L*) and cutting speed (*v_c_*) on the values of cutting forces (*Fp_avg*, *Ff_avg*, *Fc_avg*), wear of the cutting edge (*VB*), and surface roughness parameters (*Ra*) was investigated. Before starting the tests, experiments were carried out to determine the wear of the cutting edge. In the statistical analysis of the test results, the model of the matching function according to Equation (1) was adopted [13].

(1) $$Y_{1}=y-\varepsilon =b_{0}x_{0}+b_{1}x_{1}+b_{2}x_{2}+b_{3}x_{3}+b_{4}x_{4}$$

In Equation (1), *Y*_1_ is the estimated response based on first order equation, and *y* is the measured parameter (e.g., roughness parameter) on a logarithmic scale, where *x*_0_ = 1 (dummy variable) and *x*_1_–*x*_4_ are the logarithmic transformations of cutting speed, feed rate, and depth of cut, respectively; *ε* is the experimental error and *b* values are the estimates of corresponding parameters.

The *S/N* (signal-to-noise) ratio analysis strategy was adopted as “the lowest-best” according to the Equation (2) [13].

(2) $$S/N=-10\times \log\left(\frac{1}{n}\overset{n}{\underset{i\;=\;1}{\sum }}y_{i}^{2}\right)$$

where *y_i_* is the respective characteristic and *n* is the number of observations.

## 3. Results and Discussion

The research was carried out in two stages. In the first stage, the components of the cutting forces were measured (main cutting *F_c_*, feed *F_f_*, and passive *F_p_*), as well as the surface roughness of the workpiece made of sintered nickel-cobalt alloy powder as a function of the flank wear of the cutting edge *VB*. Tool wear measurements were carried out after the cutting tool had performed a turning operation of the multiple of the section *L* = 9 mm. In the next stage, in accordance with the adopted research plan, the influence of the cutting length *L* and cutting speed *v_c_* on the values of the edge wear *VB*, the cutting component *F_c_*, and the arithmetic mean value of surface roughness *Ra* were analyzed. Moreover, measurements of the geometrical dimensions of the chips were carried out, along with the analysis of the results. The average values for all analyzed parameters were determined based on 3–5 repetitions of measurements for each sample.

Table 5, Table 6 and Table 7 present selected results of the measurement of the wear of the cutting edge *VB*, the components of the total cutting force (*F_c_*, *F_f_*, *F_p_*), the and surface roughness parameter *Ra,* as a function of cutting length *L* for the cutting parameter values used in the tests (*a_p_* = 0.5 mm, *f* = 0.048 mm/rev).

**Figure 5:**
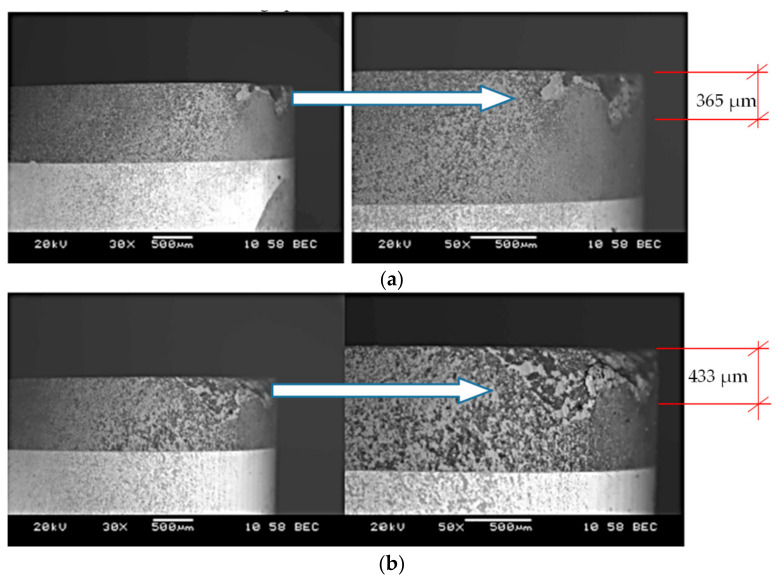
Sample photos of cutting edge wear on the cutting insert flank for (**a**) *v_c_* = 160 m/min; *f* = 0.048 mm/rev; *a_p_* = 0.5 mm, (**b**) *v_c_* = 200 m/min; *f* = 0.048 mm/rev; *a_p_* = 0.5 mm.

**Figure 6:**
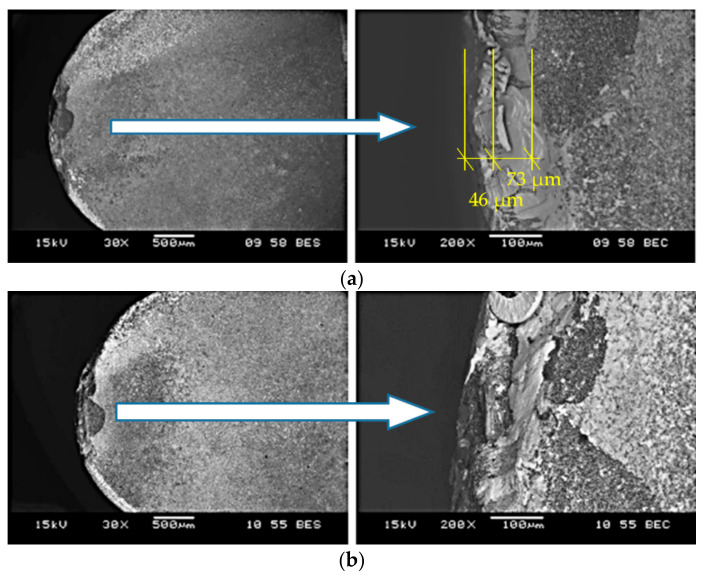
Sample photos of the cutting edge wear on the rake surface of the cutting insert (**a**) *v_c_* = 160 m/min; *f* = 0.048 mm/rev; *a_p_* = 0.5 mm, (**b**) *v_c_* = 200 m/min; *f* = 0.048 mm/rev; *a_p_* = 0.5 mm.

**Figure 7:**
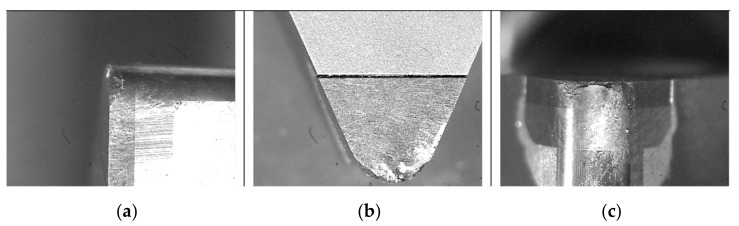
Sample photos of the cutting edge wear in the side (**a**), top (**b**) and front (**c**) view of the cutting insert for *v_c_* = 180 m/min (cast material).

**Figure 8:**
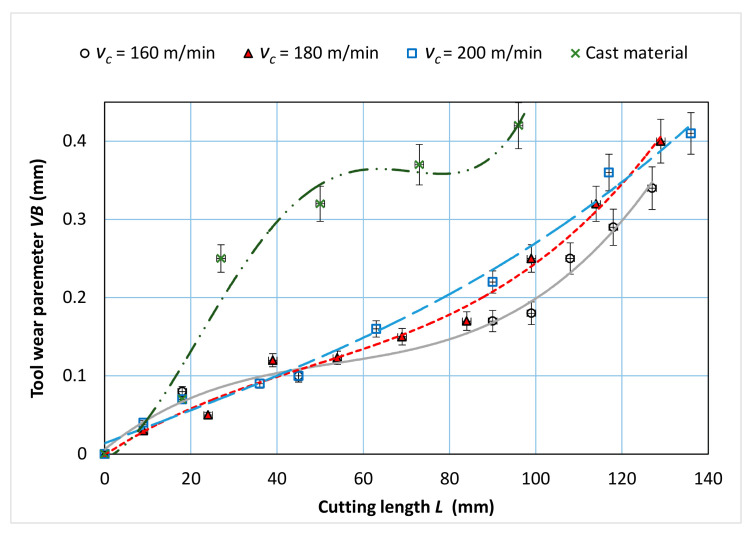
Tool wear characteristic as a function of the turning path *L* for the turning of sintered nickel-cobalt alloy with parameters: *f* = 0.048 mm/rev; *a_p_* = 0.5 mm.

Figure 5 and Figure 6 show sample photographs of the cutting edge wear used in the machining of the sintered material. To compare, Figure 7 shows an example of the cutting edge wear during machining of the cast alloy. Figure 8 shows the cutting edge wear characteristics for different cutting speeds.

SEM images of the surfaces of the cutting inserts which were used in the cutting tests of sintered material (Figure 5 and Figure 6) and cast material (Figure 7) show the wear of the cutting edge as abrasions on the surface after cutting with different cutting parameters. Significant chipping of the cutting edge material was observed on the surface, which is a common phenomenon for ceramic materials. The extent of the tool wear may have been influenced by the smaller grain size in the investigated sintered material. Based on the obtained results, the *VB* parameter characteristics were determined as a function of the cutting length (Figure 8). The presented cutting edge wear characteristics are typical, with visible preliminary lapping, then stable wear and a rapid increase in wear. In the comparison of tool wear for the machining of sintered and cast material (Figure 8), accelerated tool wear was observed, as well as a shorter tool life for machining the cast alloy. The analysis of the chemical composition showed the presence of an accumulation of the processed material in the form of elements such as Ni, Mo, Co, Cr, Ti, Al, Si, and others in trace amounts (Fe, Nb). This was also confirmed from the images of the microstructures of the rake surface and the distribution of surface element concentrations, the so called mapping.

**Figure 9:**
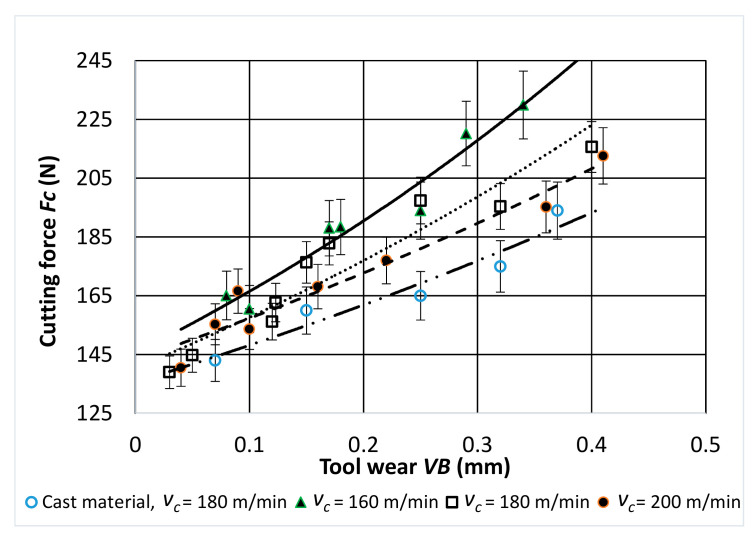
Characteristic of the *Fc* component of the total cutting force as a function of cutting edge wear *VB* for: *f* = 0.048 mm/rev; *a_p_* = 0.5 mm.

**Figure 10:**
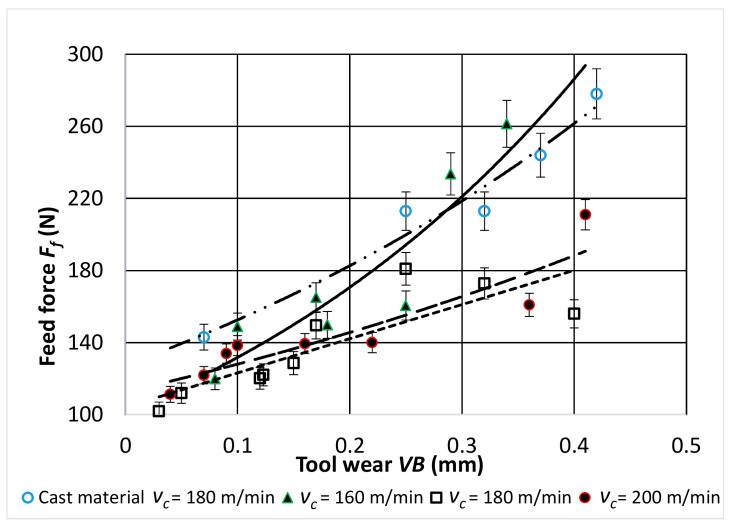
Characteristic of the *F_f_* component of the total cutting force as a function of cutting edge wear *VB* for: *f* = 0.048 mm/rev; *a_p_* = 0.5 mm.

**Figure 11:**
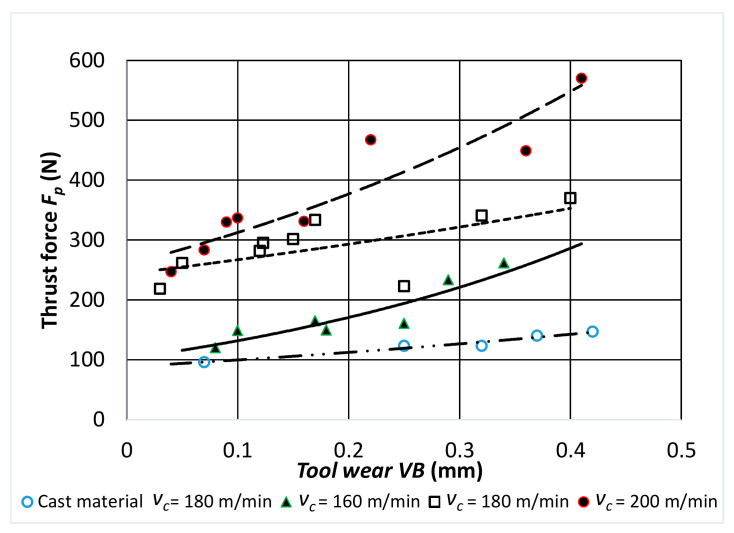
Characteristic of the *F_p_* component of the total cutting force as a function of cutting edge wear *VB* for: *f* = 0.048 mm/rev; *a_p_* = 0.5 mm.

Figure 9, Figure 10 and Figure 11 show the characteristics of the components of the total cutting force as a function of the tool wear *VB* for turning with a different value of the sintered alloy cutting speed.

A stable increase in all the values of the components of total cutting force during cutting was observed for all cutting speeds used in the tests. The highest values were obtained for the passive component *F_p_*. During the tool operation, and thus with the increasing wear of the cutting edge, the values of the passive component were about 2–2.5 times higher than the values of the other component forces.

In the next step, an ANOVA (analysis of variance) analysis of the results was carried out using the Taguchi method for three variables: cutting edge wear *VB*, surface roughness *R_a_*, and the main cutting force *F_c_*. The research plan according to the Taguchi method consisted of 18 test systems (according to Table 4). In each test system, the mean value of the analyzed variable was determined based on three measurements. Table 8 presents the results of measurements of the values of the analyzed variables.

**Figure 12:**
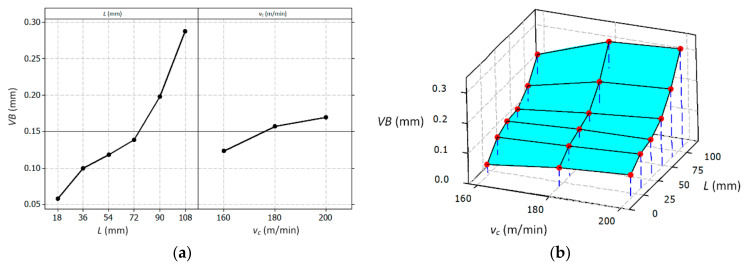
Graphic presentation of the influence of individual variables on the mean value of the *VB* parameter, main effect plot (**a**), surface plot (**b**).

**Figure 13:**
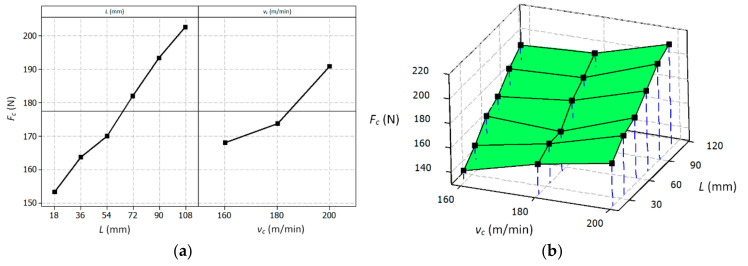
Graphic presentation of the influence of individual variables on the mean value of the total cutting force *Fc*, main effect plot (**a**), surface plot (**b**).

Figure 12 shows the influence of the variables (i.e., cutting length *L* and cutting speed *v_c_*) on the tool wear described by the *VB* parameter. Likewise, Figure 13 shows the effect of the variables on the cutting force *F_c_*.

Table 9 and Table 10 show the results of the ANOVA statistical analysis of the test results where: DF—degrees of freedom, Seq SS—sums of squares, Adj SS—adjusted sums of squares, Adj MS—adjusted means squares, P—probability of obtaining a test statistic, F—significance factor, R-Sq—coefficient of determination.

Equations (3) and (4) enable calculating the determined mathematical dependencies for cutting edge wear and the main cutting force as a function of the cutting length and cutting speed.

(3) $$VB\left(L,v_{c}\right)=-0.204-0.00232\times L+0.00115\times v_{C}$$

(4) $$F_{c}\left(L,v_{c}\right)=40+0.552\times L+0.571\times v_{C}$$

**Figure 14:**
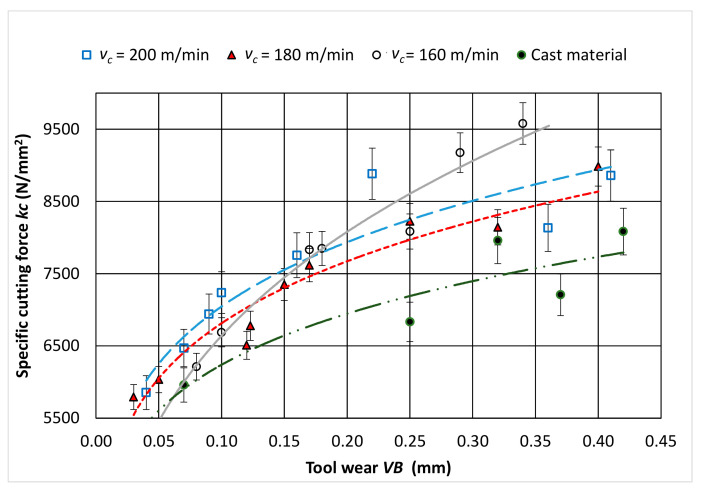
Relationship between the specific cutting force *k_c_* as a function of wear of the cutting tool *VB.*

**Figure 15:**
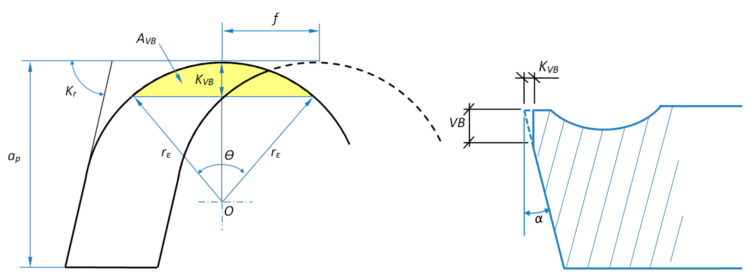
Diagram of the change of cross-section area of the cutting layer resulting from the wear of the cutting tool.

Based on the analysis of the results, it can be concluded that the change in the value of the cutting speed *v_c_* significantly affects the value of tool wear *VB* and the value of the cutting force *F_c_*. The cutting length *L* significantly affects the values of the *VB* parameter and the tangential component *F_c_*, which is the result of the progressing wear of the tool. Figure 14 shows the effect of the wear of the cutting edge on the value of the surface specific cutting force *k_c_*. It was observed that for a cutting edge wear in the range of 0.15 > *VB* > 0.35 mm, the specific cutting force values stabilized and reached the values of about *k_c_* = 8000 N/mm^2^, despite the increasing values of the cutting force *F_c_*. For wear of *VB* < 0.15 mm, a faster increase in the value of *k_c_* was visible. The reason may have been a change in the value of the cross-section of the cutting layer *A_D_* resulting from the progressing wear of the cutting edge *VB*. Figure 15 shows schematically the change of the nominal cross-sectional area *A_D_* in a perpendicular view to the rake and flank surface. The surface specific cutting force is described by the Equation (5) [41].

(5) $$k_{c}=\frac{F_{c}}{A_{D}}=\frac{F_{c}}{f\;\times \;a_{p}},\;(N/{\mathrm{mm}}^{2})$$

where *F_c_* is the main cutting force and *A_D_* is the nominal cross-section of the cutting layer.

*VB* wear on the flank surface translates into visible wear on the rake surface *K_VB_* (examples are shown in Figure 5 and Figure 6). The *K_VB_* value can be calculated approximately based on the Equation (6).

(6) $$K_{VB}=VB\times \tan\alpha ,\;(\mathrm{mm})$$

The value of the surface of the specific cutting force *k_c_* is:

(7) $$k_{c}=\frac{F_{c}}{A_{D}-A_{VB}},\;(N/{\mathrm{mm}}^{2})$$

where *A_VB_* is approximately a section of a circle and is:

(8) $$A_{VB}=\frac{r_{\varepsilon }^{2}}{2}\times \left(\theta -\sin\theta \right),\;({\mathrm{mm}}^{2})$$

During the tests, chipping of the cutting edge material on the rake surface was also observed. This phenomenon can also affect the value of the cross-section of the cutting layer during turning. For machining of cast alloy, specific cutting force values increase linearly with tool wear over time. The obtained values are, on average, about 1000 N/mm^2^ lower than the specific cutting force of the sintered alloy.

**Figure 16:**
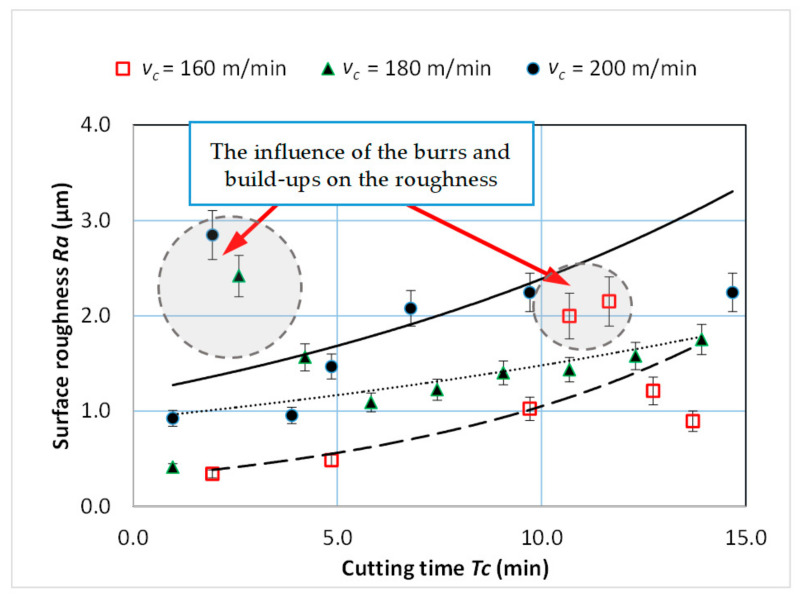
Characteristic of surface roughness *Ra* as a function of turning time for *f* = 0.048 mm/rev; *a_p_* = 0.5 mm.

**Figure 17:**
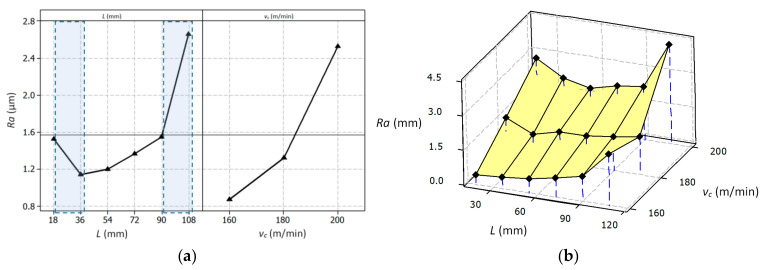
Graphic representation of the influence of particular variables on the mean value of the *Ra* parameter, main effect plot (**a**), surface plot (**b**).

Figure 16 shows the curve of the surface roughness *R_a_* as a function of the cutting time *Tc* and in time of increasing wear of the cutting edge. In the initial phase of the operation (for a new tool) and in the final phase of the cutting tool’s operation (for wear *VB* > 0.2 mm), a temporary increase in the *R_a_* parameter was observed. These changes may be caused by local deformations on the machined surface. Figure 17 shows the influence of the analyzed variables on the surface roughness *R_a_* values. The results of the statistical analysis showed a significant impact of the cutting length *L* and the cutting speed *v_c_* on the surface roughness *R_a_* values.

Table 11 shows the ANOVA results for the *R_a_* parameter. The relationship of *R_a_* as a function of the cutting length and cutting speed is presented in Equation (9).

(9) $$R_{a}\left(L,v_{b}\right)=23.847-0.024\times L-0.292\times v_{C}+0.0044\times L^{2}+0.00095\times v_{c}^{2}-0.0000113\times L\times v_{C}$$

**Figure 18:**
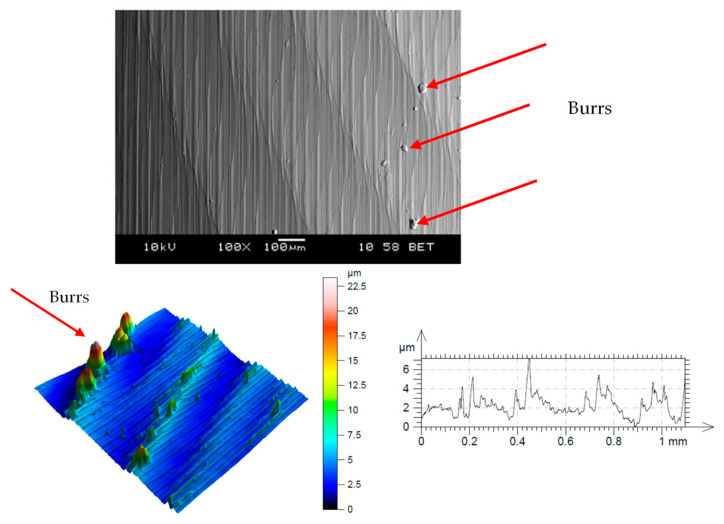
Microscopic image and topography of the machined surface with the *R_a_* characteristic (sintered nickel-cobalt alloy, *VB* < 0.2 mm, *v_c_* = 160 m/min, *a_p_* = 0.5 mm).

**Figure 19:**
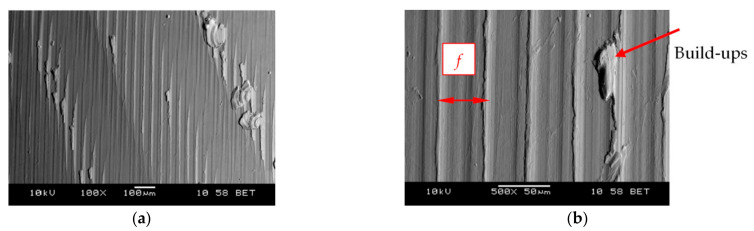
Microscopic image of the machined surface with visible deformations (build-ups); (sintered nickel-cobalt alloy, *VB* = 0.35 mm): (**a**) magnification 100×, (**b**) magnification 500×; *f*—feed.

**Figure 20:**
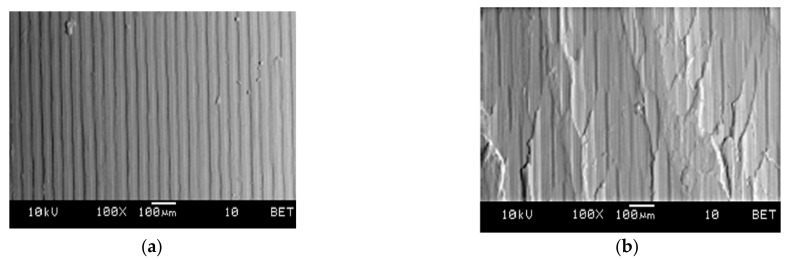
Microscopic image of the machined surface (cast alloy) (**a**) machining with a new tool, (**b**) machining with a worn tool *VB* = 0.3 mm; *v_c_* = 160 m/min, *a_p_* = 0.5 mm.

Microscopic analysis and surface topography measurements showed a changeable nature of the surface roughness as a function of the cutting length *L*, and thus the progressing wear of the cutting tool *VB*. In the initial period of the cutting edge operation, a temporary increase in the *R_a_* surface roughness value was observed. The microscopic analysis of the machined surface revealed that burrs on the machined surface were observed after a short time of tool operation (lapping the cutting edge). This may cause an increase in the value of *R_a_* parameter. Figure 18 shows examples of micrographs of the machined surface, as well as the surface topography and the *R_a_* roughness characteristic. For tool wear *VB* > 0.2 mm, workpiece build-ups and deformations were visible at the top, along the machining paths after the cutting edge had operated. Figure 19 shows examples of microscopic images with visible deformations. No such deformation was observed when cutting cast alloy. Figure 20 shows examples of microscopic images and surface topography measurements after processing the cast alloy.

**Figure 21:**
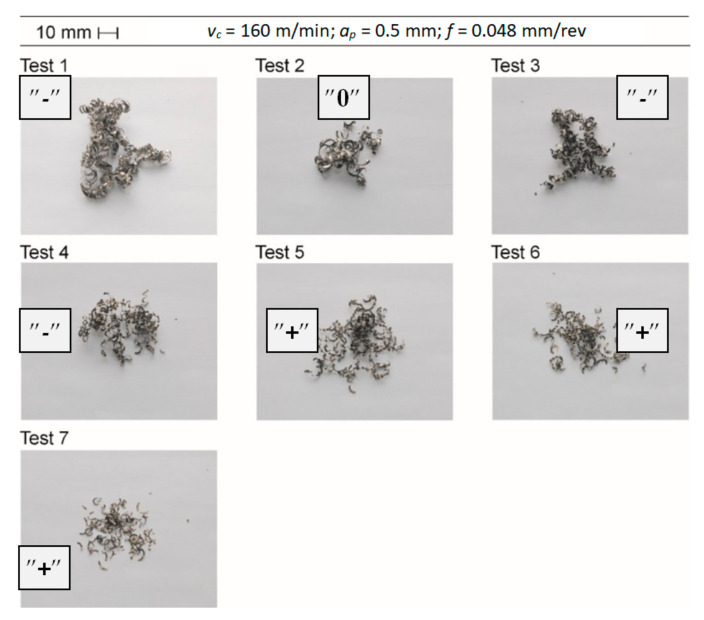
Photographs of chips for *v_c_* = 160 m/min (change of chip form), “-” = unacceptable chips with a length of more than 150 mm; “0” = acceptable chips with a length of 20–150 mm, and favorable chips “+” = with a length of up to 20 mm.

During the tests, chips were observed with a segment structure. Chip analysis showed that the chip form changed with the progressing wear of the cutting tool for all applied cutting speeds. A change in the form of chips from continuous and entangled to short and arched was observed. The probable cause of the form change was the change in the cross-section of the cutting layer resulting from the progressing wear of the cutting edge. Figure 21 shows photos of chips obtained in subsequent cutting tests.

**Figure 22:**
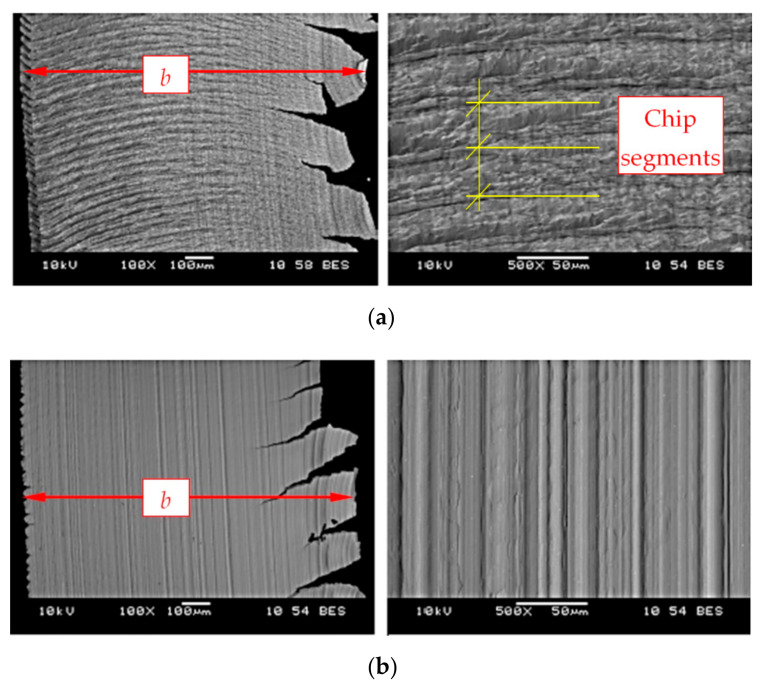
Microscopic images of the surface inside (**a**) and outside (**b**) of the chips; *v_c_* = 160 m/min, *a_p_* = 0.5 mm, *b*—chip width.

The chips were subjected to microscopic examination of the microstructure. The inside of the chips, which adhered to the rake surface, and the outside of the resulting chips were analyzed. Figure 22 shows exemplary images for microscopic chip analysis. Chip cracks were observed for the progressing wear of the cutting edge. The chips from cutting with tools with new edges, regardless of the used cutting speed *v_c_*, were silver. On the other hand, chips from cutting with worn tools presented a golden yellow color, the intensity of which increased with the increase in the cutting speed. Presumably, this could have been caused by the temperature increase in the cutting zone with the cutting speed.

**Figure 23:**
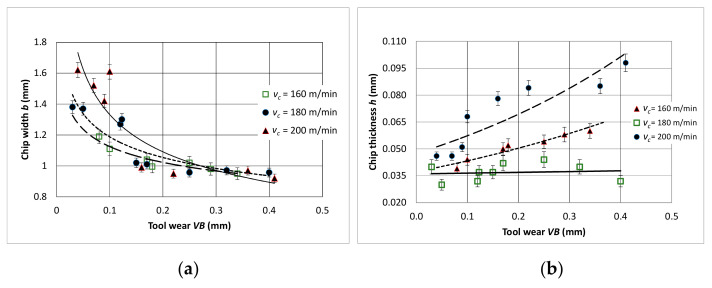

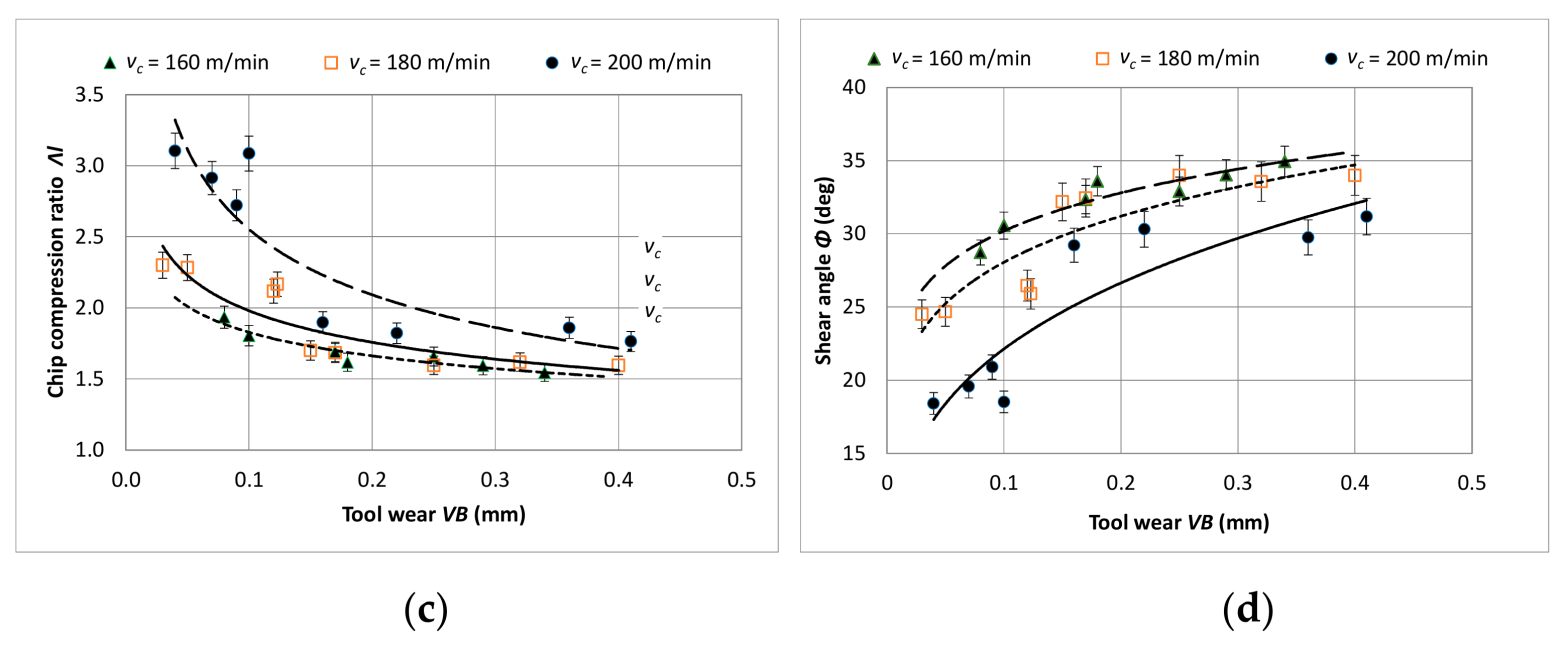
Geometric dimensions of the chips in relation to the wear of the cutting edge (**a**) chip width, (**b**) chip thickness, (**c**) chip compression ratio, (**d**) shear angle; *a_p_* = 0.5 mm.

Based on the measurements of the geometrical dimensions of the chips obtained in the cutting tests, the relationships between the dimensions of the width *h* and the thickness *b* of the chips, chip compression ratio *Λl,* and the shear angle *Φ* were determined (Figure 23). A decrease in chip width *b* was observed with a simultaneous increase in chip thickness *h* upon the progressive wear of the tool *VB*. For *VB* > 0.4 mm, the values of the chip compression ratio for all tested cutting speeds reached the value of *Λl* = 1.5. The calculated value of the shear angle was in the range of *Φ* = 18–35 degree.

## 4. Conclusions

Based on the results and analyses, the following conclusions can be formulated:

- In the article the characteristics of the machining process were presented for the parts made of sintered nickel-cobalt alloy while using CBN tools.
- During longitudinal turning of the sintered nickel-cobalt alloy, tool wear was observed. The wear on the rake face in the form of cracks and chipping material was determined. The analysis of the research results showed a significant influence of the cutting edge wear on the values of the cutting forces (*F_c_*, *F_p_*, *F_f_*), specific cutting force *kc*, surface roughness *Ra,* and chip form.
- In case of the tool wear in the range of *VB* = 0–0.2 mm, the specific cutting force values increased slightly and reached similar values equal to *k_c_* = 5500–7500 N/mm^2^. In turn, for the tool wear *VB* > 0.2 mm, the specific cutting force values increased significantly and they were characterized by a large scatter. The probable cause of this phenomenon was the change of the cross-section of the cutting layer with time due to the tool wear, as well as the change of the nature of the wear on the insert rake face: the observed chipping.
- The tool wear when machining the cast nickel-cobalt alloy was significantly higher and the tool life was shorter. Moreover, the specific cutting force values were on average lower by 1000 N/mm^2^ than the values for turning a sintered nickel-cobalt alloy.
- The microscopic analyses of the machined surface showed visible deformations, deposits, and burrs that could significantly affect the parameter values describing the surface roughness (such as *Ra*). The deterioration in the quality of the surface finish resulted from the significant tool wear. The analysis of the results showed that the surface parameter *Ra* was higher at the beginning of the tool work, and it increased significantly for the tool wear *VB* > 0.3 mm.
- The research showed the influence of the tool wear on the geometrical dimensions of the chips, and the relationships characterizing the process, e.g., the shear angle and the chip compression ratio. A change of the chip form as a function of the cutting edge wear was indicated.

The following general recommendation for the machining of sintered nickel-cobalt alloy is presented: in order to reduce the cutting forces, as well as the surface roughness parameter *Ra,* the machining should be carried out with a tool with wear not greater than *VB* = 0.2 mm.

## Figures and Tables

**Table 1:** Average grain size of the materials calculated based on the secant method.

Nickel-Cobalt Alloy	Average Grain Size (µm)	
	At the Edge of the Sample	In the Middle of the Sample
Sintered	3.38 ± 0.2	3.22 ± 0.1
Cast	34.8 ± 1.9	

**Table 2:** Chemical composition of tested alloy (%).

Ni	Co	Cr	Mo	Ti	Si	Al
57.4	20.6	13.5	2.6	2.7	0.9	2.3

**Table 3:** Tool geometry.

Main Cutting Edge Angle *Κr*	Cutting Edge Inclination Angle *λ*	Rake Angle *α*
95°	0°	−5°

**Table 4:** Research plan based on the Taguchi method.

Number	A	B	*L* (mm)	*v_c_* (m/min)
1	1	1	18	160
2	1	2	18	180
3	1	3	18	200
4	2	1	36	160
5	2	2	36	180
6	2	3	36	200
7	3	1	54	160
8	3	2	54	180
9	3	3	54	200
10	4	1	72	160
11	4	2	72	180
12	4	3	72	200
13	5	1	90	160
14	5	2	90	180
15	5	3	90	200
16	6	1	108	160
17	6	2	108	180
18	6	3	108	200

**Table 5:** Summary of the results of the measurements of the cutting edge wear, the values of the total cutting force components, and *Ra* arithmetic mean value of surface roughness for the case of turning with a speed *v_c_* = 160 m/min.

Test Number	*L*	*v_c_*	*a_p_*	*f*	*F_p_avg_*	*F_f_avg_*	*F_c_avg_*	*Ra_avg*	*VB_max_*
	(mm)	(m/min)	(mm)	(mm/rev)	(N)	(N)	(N)	(µm)	(mm)
1	18	160	0.5	0.048	300.5	119.9	140.0	0.34	0.035
2	45	160	0.5	0.048	238.2	148.9	160.5	0.48	0.10
3	90	160	0.5	0.048	389.9	165.0	185.0	1.02	0.16
4	99	160	0.5	0.048	379.6	149.8	188.4	2.00	0.18
5	108	160	0.5	0.048	387.4	160.6	195.0	2.15	0.23
6	118	160	0.5	0.048	499.1	233.6	220.2	1.21	0.29
7	127	160	0.5	0.048	468.6	261.4	229.9	0.89	0.34

**Table 6:** Summary of the results of measurements of the cutting edge wear, the values of the components of the total cutting force, and the Ra arithmetic mean value of surface roughness for the case of turning with a speed vc = 180 m/min.

Test Number	*L*	*v_c_*	*a_p_*	*f*	*F_p_avg_*	*F_f_avg_*	*F_c_avg_*	*Ra_avg*	*VB_max_*
	(mm)	(m/min)	(mm)	(mm/rev)	(N)	(N)	(N)	(µm)	(mm)
1	9	180	0.5	0.048	218.6	101.9	139.0	0.41	0.03
2	18	180	0.5	0.048	261.5	111.9	155.0	1.50	0.06
3	27	180	0.5	0.048	281.6	120.2	151.3	2.06	0.11
4	36	180	0.5	0.048	295.1	122.1	156.2	1.56	0.12
5	45	180	0.5	0.048	301.6	128.7	162.7	1.09	0.123
6	54	180	0.5	0.048	333.7	149.6	176.4	1.22	0.125
7	63	180	0.5	0.048	222.6	180.9	182.8	1.40	0.13
8	72	180	0.5	0.048	340.7	172.9	197.4	1.43	0.14
9	81	180	0.5	0.048	370.0	156.0	195.4	1.58	0.32
10	90	180	0.5	0.048	380.4	178.5	215.6	1.75	0.21

**Table 7:** Summary of the results of measurements of the cutting edge wear, the values of the components of the total cutting force, and the *Ra* arithmetic mean value of surface roughness for the case of turning with a speed *v_c_* = 200 m/min.

Test Number	*L*	*v_c_*	*a_p_*	*f*	*F_p_*	*F_f_*	*F_c_*	*Ra_avg*	*VB_max_*
	mm	m/min	mm	mm/rev	N	N	N	µm	mm
1	9	200	0.5	0.048	246.8	111.3	140.5	0.92	0.04
2	18	200	0.5	0.048	283.1	121.9	155.3	2.85	0.07
3	36	200	0.5	0.048	329.9	134.0	166.6	2.05	0.09
4	45	200	0.5	0.048	336.7	138.4	173.7	1.47	0.10
5	63	200	0.5	0.048	331.0	139.4	186.1	2.08	0.16
6	90	200	0.5	0.048	467.0	140.0	213.2	2.24	0.22
7	117	200	0.5	0.048	449.0	160.9	195.2	4.96	0.36
8	135	200	0.5	0.048	570.0	211.0	212.6	2.24	0.41

**Table 8:** The layout of the research plan with the mean values of the analyzed variables.

A	B	*L* (mm)	*v_c_* (m/min)	*VB__avg_* (mm)	*Ra__avg_* (µm)	*F_c_avg_* (N)
1	1	18	160	0.035	0.34	140
1	2	18	180	0.062	1.50	155
1	3	18	200	0.076	2.75	165
2	1	36	160	0.090	0.42	151
2	2	36	180	0.100	0.95	162
2	3	36	200	0.110	2.06	178
3	1	54	160	0.110	0.54	165
3	2	54	180	0.120	1.25	162
3	3	54	200	0.125	1.81	183
4	1	72	160	0.115	0.75	172
4	2	72	180	0.140	1.25	178
4	3	72	200	0.160	2.10	196
5	1	90	160	0.160	1.02	185
5	2	90	180	0.210	1.40	187
5	3	90	200	0.223	2.22	208
6	1	108	160	0.230	2.15	195
6	2	108	180	0.310	1.58	198
6	3	108	200	0.323	4.24	215

**Table 9:** ANOVA statistical analysis for *VB* (mm).

Source	DF	Seq SS	Adj SS	Adj MS	F	P
*L* (mm)	5	0.100166	0.100166	0.020033	75.73	0.0
*v_c_* (m/min)	2	0.006842	0.006842	0.003421	12.93	0.002
Residual Error	10	0.002645	0.002645	0.000265	-	-
Total	17	0.109653	-	-	-	-
R-Sq = 98.3%	-	-	-	-	-	-

**Table 10:** ANOVA statistical analysis for *Fc* (mm).

Source	DF	Seq SS	Adj SS	Adj MS	F	P
*L* (mm)	5	5207.8	5207.8	1041.57	789.53	0.0
*v_c_* (m/min)	2	1696.3	1696.3	848.17	72.91	0.0
Residual Error	10	116.3	116.3	11.63	-	-
Total	17	7020.5	-	-	-	-
R-Sq = 98.3%	-	-	-	-	-	-

**Table 11:** ANOVA statistical analysis for *Ra* (µm).

Source	DF	Seq SS	Adj SS	Adj MS	F	P
*L* (mm)	5	4.630	4.630	0.9259	3.91	0.016
*v_c_* (m/min)	2	8.839	8.839	4.4197	23.46	0.0
Residual Error	10	1.884	1.884	0.1884	-	-
Total	17	15.353	-	-	-	-
R-Sq = 87.7%	-	-	-	-	-	-

## Data Availability

The data presented in this study are available on request from the corresponding author. The data are not publicly available due to privacy.

## Abbreviations

*Κr*	Main cutting edge angle (°)
*α*	Rake angle (°)
*λ*	Cutting edge inclination angle (°)
*Ra*	Arithmetic mean value of surface roughness (µm)
*F_c_*	Cutting force (N)
*F_p_*	Thrust force (N)
*F_f_*	Feed force (N)
*v_c_*	Cutting speed (m/min)
*k_c_*	Specific cutting force (N/mm^2^)
